# Analysis of clinical characteristics and prognosis of 68 patients with primary pulmonary choriocarcinoma

**DOI:** 10.1186/s12890-023-02368-w

**Published:** 2023-03-08

**Authors:** Xinyi Cao, Honglin Feng, Shengming Liu, Li Chen

**Affiliations:** Department of Pulmonary and Critical Care Medicine, The First Affiliated Hospital of Jinan University, Guangzhou, China

**Keywords:** Choriocarcinoma, Nongestational, Lung neoplasms, Retrospective studies, Prognosis

## Abstract

**Background:**

Primary pulmonary choriocarcinoma (PPC) is a highly malignant intrapulmonary tumor with a notorious prognosis. Few clinical studies have been undertaken to investigate the clinical characteristics and prognosis of PPC.

**Material and methods:**

We systematically conducted a retrospective analysis of patients with PPC in the literature published in PubMed and CNKI databases until March 31, 2022. The primary outcome was all-cause mortality. Survival curves were depicted using the Kaplan‒Meier method and compared using the stratified log-rank test. A Cox proportional hazards model was used to estimate the prognostic factors.

**Results:**

A total of 68 patients were included, which consisted of 32 females and 36 males, with an average age of (44.5 ± 16.8) years old, ranging from 19 to 77 years. The clinical characteristics were mostly cough (49.2%), dyspnea (22.2%), hemoptysis (39.7%) and chest pain (39.7%). Kaplan‒Meier analysis showed that sex, age, hemoptysis, metastasis and treatment combining surgery with chemotherapy had a significant effect on survival. There were no effects on other outcomes. Furthermore, univariate and multivariable Cox regression analyses showed that the impact of the treatment combining surgery with chemotherapy on OS showed independent prognostic significance.

**Conclusion:**

PPC is a rare disease that lacks specific clinical features. Early diagnosis with optimal management is a significant goal. Surgery followed by adjuvant chemotherapy may be the best treatment for PPC.

**Supplementary Information:**

The online version contains supplementary material available at 10.1186/s12890-023-02368-w.

## Introduction:

Primary pulmonary choriocarcinoma (PPC) is a rare and highly malignant germ cell neoplasm that secretes human chorionic gonadotropin β-subunit (β-hCG) and arises spontaneously in the lung. This clinical entity can easily be misdiagnosed or become a delayed diagnosis due to its unspecific clinical characteristics. Although PPC is a type of choriocarcinoma, there is no established standard therapy for this disease, which is mainly administered according to the treatment guidelines for choriocarcinoma. For patients with gestational choriocarcinoma, the complete response rate of single-drug chemotherapy in low-risk patients is close to 100%, while that in high-risk patients is approximately 85% [1]. However, chemotherapy for PPC is not as effective as for gestational choriocarcinoma, and a poor prognosis has been reported for this disease. The poor prognosis is attributed to the rarity of the disease, difficulty in diagnosis and rapidly fatal natural course of PPC, which often results in a lack of optimal treatment time. Early diagnosis with optimal management is always a significant goal, which can improve the prognosis. Therefore, we conducted a retrospective analysis of 68 patients diagnosed with primary pulmonary choriocarcinoma, elucidating the clinical characteristics and prognosis, to improve the understanding and optimal management of this rare neoplasm.

## Material and methods

### Data sources and searches

We conducted a comprehensive literature search in PubMed and CNKI databases using the following search strategy: “Primary”[All Fields] AND ("choriocarcinoma"[MeSH Terms] OR "choriocarcinoma"[All Fields]) AND ("lung" [MeSH Terms] OR "lung" [All Fields] "OR "Pulmonary" [MeSH Terms] OR " Pulmonary " [All Fields]). The article type and language were not restricted during the initial search.

### Study selection

We included all patients diagnosed with PPC who were published in the literature until March 31, 2022. The inclusion criteria included the following: (1) no history of gynecologic cancers; (2) isolated or predominant lesion detected in the lung; (3) elevated levels of β-human chorionic gonadotropin (β-hCG) in the blood or urine, which decreased after surgery or chemotherapy; (4) thorough examination of reproductive organs and midline structures to rule out potential primary choriocarcinoma in the gonads or tumor metastasis to the lung; and (5) histopathological confirmation of choriocarcinoma [2–4]. The exclusion criteria were as follows: (1) pulmonary metastasis of gestational choriocarcinoma; (2) primary nontrophoblast tumor of the lung, such as giant cell carcinoma of the lung; (3) < 1 year old; and (4) incomplete clinical data, such as for articles in which the full text could not be obtained and there was no survival time described, etc.

### Data extraction and quality assessment

For each included study, 2 reviewers extracted data in a predefined Excel format independently using the criteria described in "Study Selection". Discrepancies were resolved by discussion with the research team, as necessary. The extracted data included (1) first author and year of publication, (2) region, (3) sex, (4) age, (5) initial symptoms, (6) lesion area, (7) tumor size, (8) metastasis, (9) pathological type, (10) therapy, and (11) survival time.

### Data synthesis and analysis

The primary endpoint was overall survival (OS). All eligible patients were included in the analysis of overall survival. OS was defined as the period from diagnosis to death from any cause or the last follow-up time. Survival curves were depicted using the Kaplan‒Meier method and compared using the stratified log-rank test. A Cox proportional hazards model was used to estimate the simultaneous effects of prognostic factors on survival. Statistical analyses to identify risk factors were performed using SPSS 25.0 statistical software. Differences were considered to be statistically significant when the *P* value was 0.05 or less. All statistical tests were two-sided.

## Results:

### Description of included studies

**Fig. 1:**
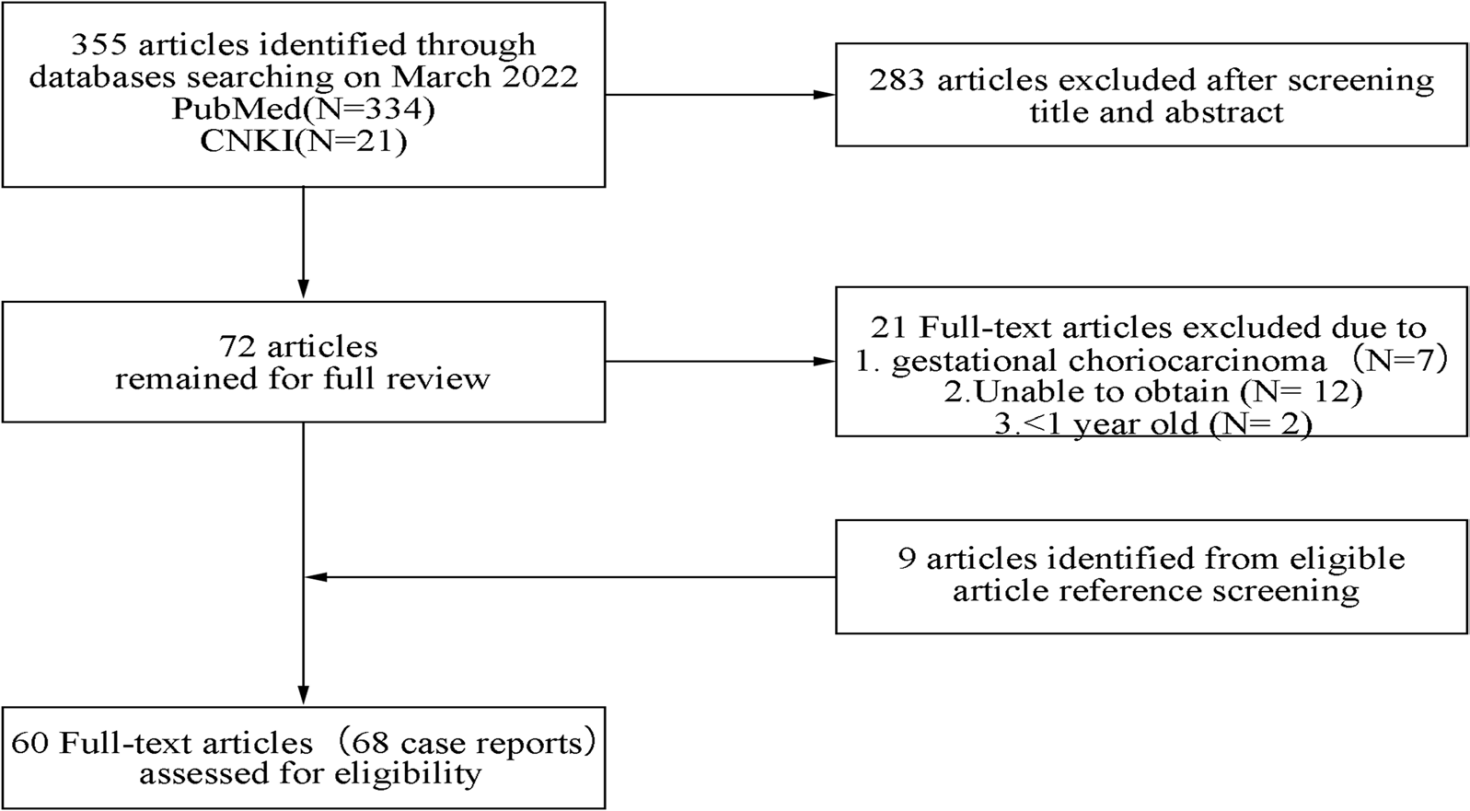
The flow of patient screening and enrollment

The search strategy identified 355 articles through databases using the criteria described in the methods. Following screening the titles and abstracts, 72 abstracts were identified, which were read in full. Of these, 9 did not meet the inclusion criteria, 12 were not available in full text, and 9 were identified from eligible article reference screening. Finally, 60 articles met the prespecified criteria for inclusion (Fig. 1). Through reading, we collected 68 cases of primary choriocarcinoma originating in the lung (Additional file 1: Table S1).

### Clinical characteristics of the patients

This study consisted of 36 males and 32 females with an average age of 44.5 ± 16.8 years (range 19 to 77). At the time of admission to the hospital, 63 patients (93%) developed symptoms, including cough (49.2%), dyspnea (22.2%), hemoptysis (39.7%), chest pain (39.7%), etc. Signs of feminization, such as gynecomastia, may also be observed in some male patients, but this is uncommon. The baseline clinical characteristics of the patients are summarized in Table 1. In the OP + CT cohort, most patients who underwent surgery received either radical lobectomy (62.5%) or palliative surgery (37.5%). Regional lymph node dissection (LNE) was performed in 6 (25.0%) patients. The chemotherapy regimens were form treating gestational trophoblastic neoplasia, and the chemotherapy regimens mostly used the PEB regimen, EMA-CO regimen, EMA-EP regimen and so on.

**Table 1:** Summary of demographic and clinical characteristics

Characteristics	N = 68	%
*Age (years)*		
< 40	31	45.6
≥ 40	37	54.4
*Sex*		
F	32	47.1
M	36	52.9
*Region*		
Asian	41	65.1
Others	22	34.9
*Initial symptoms*		
Cough	31	45.6
Dyspnea	14	20.6
Hemoptysis	25	36.8
Chest pain	25	36.8
Asymptomatic	5	7.4
*Lesion location*		
L	17	25.0
R	41	60.3
Bilateral	10	14.7
*Tumor size, cm*		
≤ 5	28	52.8
> 5	25	47.2
*Metastasis site*		
CNS	17	56.7
Lung	17	56.7
*Pathological type*		
Complex	16	23.5
Simple	52	76.5
*Treatment*		
OP	16	23.5
CT	14	20.6
OP, CT	24	35.3
OP, CT, RT	7	10.3
Others	7	10.3

*M* male, *F* female, *R* right lung, *L* left lung, *CNS* central nervous system, *OP* operation, *CT* chemotherapy, *RT* radiotherapy

Only two patients underwent genetic mutation analysis using clinical cancer genome sequencing. Unfortunately, for several reasons, none of the patients received immune checkpoint inhibitors.

### Overall survival

**Fig. 2:**
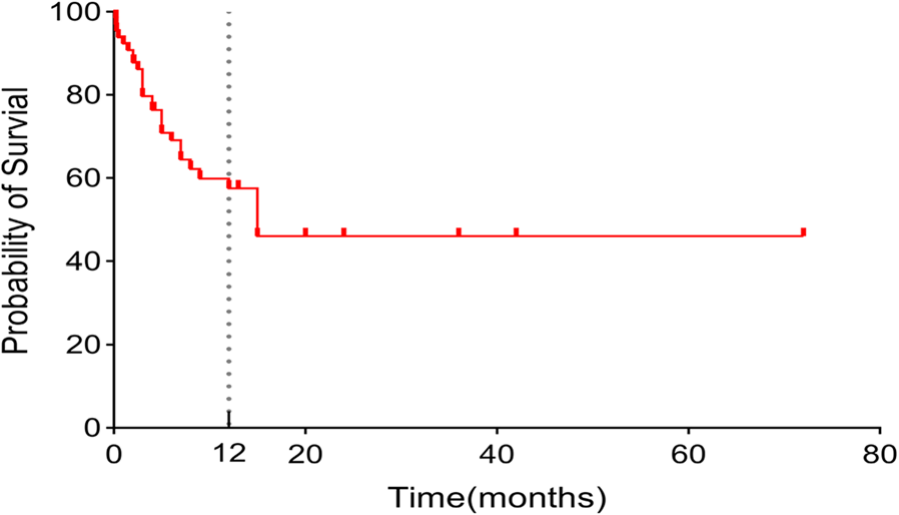
Kaplan‒Meier curve for overall survival (N = 68)

Figure 2 illustrates that the median survival time was 15 months in the 68 patients, and the 1-year overall survival rate was 55.7%.

**Fig. 3:**
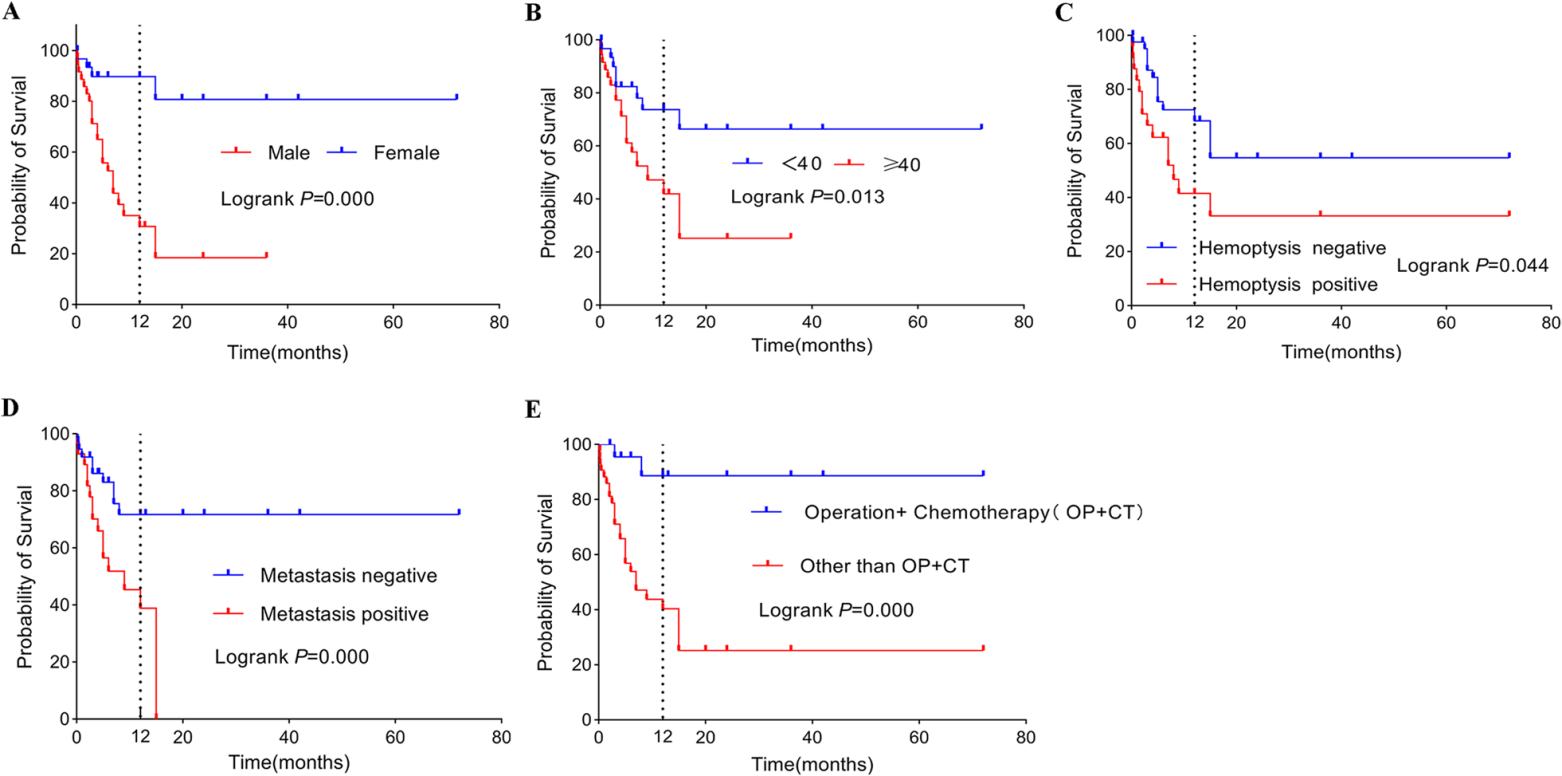
The probability of overall survival in PPC was calculated using the Kaplan‒Meier method stratified by (**A**) sex, (**B**) age, (**C**) hemoptysis, (**D**) metastasis and (**E**) treatment

As shown in Fig. 3, the probability of overall survival in the PPC patients was calculated using the Kaplan‒Meier method stratified by sex, age, hemoptysis, metastasis, and treatment. In the comparison of overall survival between sexes, a significant difference (log-rank test, *P* = 0.000) was observed, with women showing a 1-year survival rate of 78.3% and men showing a 1-year survival rate of 29.7%.

As expected, the Kaplan‒Meier analysis showed worse OS for older patients (≥ 40 years) than for younger patients (< 40 years) (log-rank test, *P* = 0.013). The survival of patients without hemoptysis was longer than that of patients with hemoptysis (log-rank test, *P* = 0.044). Patients without metastasis had better OS than those with metastasis (log-rank test, *P* = 0.000). The results of the patients with and without treatment combining surgery with chemotherapy showed a significant difference between the two groups (log-rank test,* P* = 0.000). For other factors, there were no significant influences.

### Results of selection of predictors

Univariate Cox regression analysis of the factors related to mortality in PPC patients is shown in Table 2. Age, sex, region, metastasis, and treatment combining surgery with chemotherapy were significantly associated with the mortality of PPC patients (*P* < 0.05). After performing the enter method in multivariate Cox regression analysis, 2 predictors had independent prognostic significance: treatment combining surgery with chemotherapy (HR 0.207; 95% CI 0.045–0.958) and sex (HR 0.228; 95% CI 0.066–0.787) (Table 3).

**Table 2:** Possible factors related to the mortality of the PPC patients as shown by univariate Cox regression analysis

Characteristics	β	S.E	*P*	HR (95% CI)
Region	− 0.922	0.467	0.048	0.398 (0.159–0.994)
Sex	− 1.945	0.546	0.000	0.143 (0.049–0.417)
Age (Years)	1.003	0.427	0.019	2.728 (1.182–6.296)
Cough	0.129	0.386	0.737	1.138 (0.535–2.423)
Dyspnea	0.322	0.464	0.487	1.380 (0.556–3.423)
Chest pain	− 0.478	0.411	0.245	0.620 (0.277–1.388)
Hemoptysis	0.747	0.386	0.053	2.110 (0.991–4.493)
Size(mm)	0.670	0.434	0.123	1.954 (0.834–4.574)
Metastasis	1.323	0.418	0.002	3.755 (1.654–8.523)
Location	0.383	0.226	0.091	1.467 (0.941–2.286)
Pathologic types	0.627	0.413	0.129	1.872 (0.833–4.206)
Treatment				
OP	0.472	0.425	0.266	1.604 (0.697–3.689)
CT	0.743	0.442	0.092	2.103 (0.885–4.997)
OP + CT	− 2.265	0.736	0.002	0.104 (0.025–0.439)

*HR* hazard ratio, *CI* confidence interval, *OP* operation, *CT* chemotherapy

**Table 3:** Factors related to mortality of the PPC patients by multivariate Cox regression analysis

Characteristics	β	S.E	*P*	HR (95% CI)
Sex	− 1.478	0.632	0.019	0.228 (0.066–0.787)
Age (Years)	− 0.526	0.508	0.300	0.591 (0.218–1.598)
Region	− 0.454	0.569	0.425	0.635 (0.208–1.937)
Metastasis	0.515	0.453	0.256	1.673 (0.689–4.065)
OP + CT	− 1.575	0.781	0.044	0.207 (0.045–0.958)

*HR* hazard ratio, *CI* confidence interval, *OP* operation, *CT* chemotherapy

## Discussion

PPC is a highly malignant intrapulmonary tumor with a notorious prognosis, and its pathogenesis is poorly understood. In the literature, several theories have been postulated to explain the development of PPC: (1) origin from primordial germ cells that remain from abnormal migration during embryonic development; (2) origin from metastasis of gonadal choriocarcinoma, while the gonadal primary tumor has regressed spontaneously; or origin from a trophoblastic embolus related to the gestational event after a long period of latency; and (3) the tumor is a primary nontrophoblastic tumor of the lung, which transforms into choriocarcinoma [5–8].

The clinical characteristics of PPC vary and usually present with symptoms such as dyspnea, cough, chest pain or hemoptysis. In men, signs of feminization, such as gynecomastia, loss of libido, and testicular atrophy, may be seen, which are associated with elevations in the β-hCG levels. Although progressive elevations of β-hCG are usually beneficial in detecting choriocarcinoma lesions, β-hCG detection is often neglected in clinical practice, particularly in male patients, and this makes ectopic or primary choriocarcinoma to be often misdiagnosed or missed, which makes it difficult to make a diagnosis of PPC at an early stage.

In this study, a significant difference in overall survival time was observed between patients with and without hemoptysis (*p* = 0.044), which is probably related to the vasculophilic nature of tumors originating from trophoblastic cells. Choriocarcinoma is often perfused by fragile blood vessels, and when the tumor develops aggressive growth, it will easily break through the vessels, leading to hemorrhage [9]. When presented with hemoptysis, we should be alert for the development of metastasis since PPC has an affinity for blood vessels and is prone to hematogenous metastasis. As shown in Fig. 3, it was also observed that patients without metastasis at presentation had a longer survival than patients with metastasis, indicating that the occurrence of metastasis increases the tumor load and leads to an extremely poor prognosis, which is consistent with the previous literature [10]. However, the analysis of region and tumor size were not statistically significant, considering that it was related to the relatively small sample.

Between the sexes, this study observed a better prognostic outcome for female patients than for male patients, and the exact reason remains unclear. Snoj, Z et al. explored pregnancy events as a prognostic factor and found that women with a history of gestational events had a better prognosis than those without a history of gestational events [11]. However, in the absence of characteristic immunohistochemical features of PPC, it remains controversial whether women with pregnancy events have unique pathophysiological features or whether occult gestational choriocarcinoma with pulmonary metastasis is misdiagnosed as PPC, leading to a good survival outcome on statistical analysis. Recently, genetic examinations, such as microsatellite genotyping [12] and short tandem repeat sequencing [13], etc., have been used to distinguish gestational from nonpregnant trophoblastic tumors. Maesta et al. confirmed that the patients in both of their cases were of gestational origin [14]. Vegh et al. excluded gestational origin, despite their patient having a history of pregnancy [15]. Placental emboli of previous pregnancies may be a mechanism for the development of PPC in women with pregnancy events. Therefore, genetic examination of tissues may be necessary to eliminate confounding factors, provided that the genital tract and midline structures are thoroughly examined, but these techniques, at present, are difficult to promote widely in clinical practice.

At present, there is no established standard therapy for this disease, which is mainly administered according to the treatment guidelines for choriocarcinoma and the clinicians' experience. Due to the undifferentiated nature of the malignancy, PPC responds poorly to radiotherapy [16]. To explore the optimal treatment for PPC, a univariate and multifactorial analysis of the data collected was carried out, showing that chemotherapy alone seldom improved the prognosis of patients, but surgery followed by adjuvant chemotherapy had a remarkable effect on the prognosis and was independently prognostically significant. The results we obtained are consistent with the known fact that PPC is an undifferentiated aggressive tumor. Therefore, adjuvant chemotherapy after surgery seems to be the best treatment for early-stage patients with limited lesions.

Next-generation sequencing is being more widely used as a valuable method for finding immunotargeted therapies. Gene detection may help identify biomarkers that are prognostic or predictive and can be used for the development of novel therapies in the future. Ma, Y et al. conducted gene detection and revealed eight genetic mutations, including two targeted drug-related mutations (STK11 and SMARCA4) [17]. Onishi, I et al. performed genetic mutation analysis using clinical cancer genome sequencing, which identified an epidermal growth factor (EGFR) V774M mutation (activating mutation) on exon 20 [18]. Unfortunately, for several reasons, none of the patients received immune checkpoint inhibitors. In conclusion, the accumulation of cases and genetic analysis in the genome sequence era may help improve prognosis, which requires more scholars to investigate in-depth the associated gene mutations and signaling pathways in the future.

## Limitations

There are several limitations in this study. First, given the retrospective nature of the data collection from the PubMed and CNKI databases, inherent selection bias was inevitable. Second, the current sample may not be large enough to perform a high-quality analysis. Our results should be interpreted with caution given the limitations discussed above. Hence, more research is needed in the future to validate these results.

## Conclusion

PPC is a rare disease that lacks specific clinical features. There is no established standard therapy for this disease, and surgery followed by adjuvant chemotherapy may be the best treatment for PPC. Targeted therapy is currently a hot spot in tumor treatment, and more scholars should investigate the associated gene mutations and signaling pathways for PPC in the future to improve the prognosis of patients.

## Supplementary Information

**Additional file 1: Table S1.** Summary of the literature review reporting PPC.

## Data Availability

The data analyzed in the current study can be collected in the PubMed (https://pubmed.ncbi.nlm.nih.gov/) and CNKI (https://cnki.net/) databases, and all data are included in Additional file 1: Table S1.

## Abbreviations

CNKI: China National Knowledge Infrastructure
PPC: Primary pulmonary choriocarcinoma
OS: Overall survival
M: Male
F: Female
R: Right lung
L: Left lung
CNS: Central nervous system
HR: Hazard ratio
CI: Confidence interval
OP: Operation
CT: Chemotherapy
RT: Radiotherapy

## References

[1] Ngan HY, Seckl MJ, Berkowitz RS (2015). Update on the diagnosis and management of gestational trophoblastic disease. Int J Gynaecol Obstet.

[2] Di Crescenzo V, Laperuta P, Napolitano F, Carlomagno C, Garzi A, Vitale M (2013). An unusual case of primary choriocarcinoma of the lung. BMC Surg.

[3] Takahashi T, Kobayashi R (2016). Choriocarcinoma syndrome after resection of primary pulmonary choriocarcinoma: report of a case. Surg Case Rep.

[4] Kamata S, Sakurada A, Sato N, Noda M, Okada Y (2017). A case of primary pulmonary choriocarcinoma successfully treated by surgery. Gen Thorac Cardiovasc Surg.

[5] Serno J, Zeppernick F, Jakel J (2012). Primary pulmonary choriocarcinoma: case report and review of the literature. Gynecol Obstet Invest.

[6] Toda S, Inoue Y, Ishino T (1995). A rare case of primary pulmonary choriocarcinoma in a male: immunohistochemical detection for human chorionic gonadotropin, epidermal growth factor (EGF) and EGF-receptor. Endocr J.

[7] Adachi H, Aki T, Yoshida H, Yumoto T, Wakahara H (1989). Combined choriocarcinoma and adenocarcinoma of the lung. Acta Pathol Jpn.

[8] Aparicio J, Oltra A, Martinez-Moragon E, Llorca C, Gomez-Aldaravi L, Pastor M (1996). Extragonadal nongestational choriocarcinoma involving the lung: a report of three cases. Respiration.

[9] Kageji T, Nagahiro S, Matsuzaki K (2007). Successful neoadjuvant synchronous chemo- and radiotherapy for disseminated primary intracranial choriocarcinoma: case report. J Neurooncol.

[10] Umemori Y, Hiraki A, Aoe K (2004). Primary choriocarcinoma of the lung. Anticancer Res.

[11] Snoj Z, Kocijancic I, Skof E (2017). Primary pulmonary choriocarcinoma. Radiol Oncol.

[12] Fenichel P, Rouzier C, Butori C (2014). Extragestational betaHCG secretion due to an isolated lung epithelioid trophoblastic tumor: microsatellite genotyping of tumoral cells confirmed their placental origin and oriented specific chemotherapy. J Clin Endocrinol Metab.

[13] Buza N, Baine I, Hui P (2019). Precision genotyping diagnosis of lung tumours with trophoblastic morphology in young women. Mod Pathol.

[14] Maesta I, Leite FV, Michelin OC, Rogatto SR (2010). Primary pulmonary choriocarcinoma after human chorionic gonadotropin normalization following hydatidiform mole: a report of two cases. J Reprod Med.

[15] Vegh GL, Szigetvari I, Soltesz I (2008). Primary pulmonary choriocarcinoma: a case report. J Reprod Med.

[16] Sridhar KS, Saldana MJ, Thurer RJ, Beattie EJ (1989). Primary choriocarcinoma of the lung: report of a case treated with intensive multimodality therapy and review of the literature. J Surg Oncol.

[17] Ma Y, Wang C, Sun PL, Zhu Y, Huang ZK, Jin SX (2018). A case of male primary pulmonary choriocarcinoma. Chin Med J (Engl).

[18] Onishi I, Kirimura S, Wakejima R (2022). Primary pulmonary choriocarcinoma with a genomic sequence. Pathol Int.

