# Supplementary material for: Analysis of clinical characteristics and prognosis of 68 patients with primary pulmonary choriocarcinoma
Source: BMC Pulm Med. 2023 Mar 8;23:75. doi: 10.1186/s12890-023-02368-w (PMC9996839; doi:10.1186/s12890-023-02368-w)
Supplement: Supplementary file 1 — Additional file 1: Table S1. Summary of the literature review reporting PPC. [file 12890_2023_2368_MOESM1_ESM.docx]

Supplementary Table 1

| Author | region | Sex | Age  (year) | Initial  symptom(s) | Location | Tumor size(cm) | Metastasis | Pathological  type | Therapy | Survival  (months) |
| --- | --- | --- | --- | --- | --- | --- | --- | --- | --- | --- |
| Gerin-Lajoie L | Others | F | 29 | Cough, chest pain | RUL | 11 | lung | CC | OP | 3 |
| Cacciamani J | NA | M | 19 | Cough | RLL | 2 |  | CC | CT | 2.5 |
| Hayakawa K *et al.* | Asia | M | 45 | Gynecomastia | LUL | 7 | Lung, liver, kidney, spleen | CC | RT, Other | 5 |
| Hayakawa K *et al.* | Asia | M | 57 | Cough, gynecomastia | RLL | 10 | brain | CC | RT, Other | 12 |
| Kalla AH *et al*. | NA | M | 27 | Chest pain | RUL | 4 |  | CC | OP, CT | 24 |
| Zapatero J *et al.* | Others | M | 67 | Hemoptysis | LUL | 5 |  | CC | OP | 36 |
| Patra SB *et al.* | NA | F | 22 | Cough, dyspnea | LUL | 25 | lung, brain, spleen | CC | CT | 0.23 |
| Tanimura A *et al.* | Asia | F | 34 | Cough, chest pain | RUL | 4 |  | CC | OP, CT | 6 |
| Pushchak MJ *et al.* | NA | F | 60 | Hemoptysis | R | 11 | lung | CC | OP | 0.23 |
| Rhee YK *et al.* | Asia | F | 33 | Cough, dyspnea, weight loss | RUL, RML | NA |  | CC | CT | 2.5 |
| Endou T *et al.* | Asia | M | 37 | Gynecomastia | Bilateral | 7 | liver, lung, spleen, kidney | CC | CT | 2.5 |
| McLeod DT. | Others | F | 21 | Cough, dyspnea, hemoptysis, chest pain, weight loss, vaginal spotting | LUL | NA | brain | CC | CT | 2 |
| Sullivan LG | NA | M | 51 | Cough, weight loss | LUL | 15 | lung, brain, liver, spleen | CC | RT, Other | 0.23 |
| Adachi H *et al.* | Asia | M | 71 | Dyspnea, chest pain | RUL | 3 | lung, brain, liver, kidney, adrenal gland, spleen | CC, ADC | RT, Other | 6 |
| Sridhar KS *et al.* | Others | M | 37 | Cough, hemoptysis, chest pain, fever | RUL | 11 | brain | CC, SCC | OP, CT, RT | 15 |
| Durieu I *et al.* | Others | M | 61 | Hemoptysis, chest pain | LUL | 6 |  | CC | OP, CT, RT | 0.46 |
| Van Nostrand KM *et al.* | Others | F | 27 | Positive urine pregnancy test | RUL, RML | 2.8 |  | CC, ADC | OP, CT | 36 |
| Toda S *et al.* | Asia | M | 69 | Hemoptysis, weight loss, gynecomastia | RLL | 18 | lung, liver, adrenal gland | CC | CT | 1.5 |
| Canver CC *et al*. | Others | M | 69 | Asymptomatic | LUL | 5 |  | CC, SCC | OP, CT | 6 |

Summary of literature review reporting PPC

Supplementary Table 1 (continued)

| Author | region | Sex | Age  (year) | Initial  symptom(s) | Location | Tumor size(cm) | Metastasis | Pathological  type | Therapy | Survival  (months) |
| --- | --- | --- | --- | --- | --- | --- | --- | --- | --- | --- |
| Aparicio J *et al.* | Others | F | 29 | Cough, hemoptysis, chest pain | Bilateral | 2 |  | CC | CT | 72 |
| Aparicio J*et al.* | Others | F | 54 | Chest pain | RUL | 9 | brain, liver, kidney | CC | OP, CT, RT | 12 |
| Aparicio J *et al.* | Others | F | 57 | Cough, dyspnea, weight loss | RUL | NA | lung, adrenal gland | CC, ASC | CT | 4.2 |
| Ikura Y *et al.* | Asia | M | 60 | Asymptomatic | R | NA | lung, brain, testicle | CC, SCC | CT | 5 |
| Chen F *et al.* | Asia | M | 61 | Hemoptysis | RLL | NA | lung | CC, ADC | OP, CT | 6 |
| Okur E *et al.* | Others | M | 46 | Cough, dyspnea, chest pain, weight loss | RUL | 18 |  | CC, MGCT | OP, CT | 6 |
| Tsai JR *et al.* | Asia | M | 23 | Dyspnea, hemoptysis | Bilateral | NA |  | CC | CT | 0.27 |
| Arslanian A *et al.* | Others | F | 37 | Asymptomatic | RLL | NA |  | CC | OP, CT | 12 |
| Umemori Y *et al.* | Asia | F | 31 | Hemoptysis, chest pain | LUL | 8 |  | CC | OP, CT | 36 |
| Gang Shen *et al.* | Asia | M | 19 | Hemoptysis, chest pain, precocious puberty | Bilateral | 12 |  | CC | OP | 7 |
| Cho YJ *et al.* | Asia | F | 28 | Positive urine pregnancy test | RLL | 5 |  | CC | OP | 4 |
| Yamamoto S *et al.* | Asia | M | 77 | Hemoptysis | LUL | 5.5 | lung liver, spleen, cardiac, adrenal gland, kidney, thyroid | CC, ADC | RT, Other | 0.2 |
| Shintaku M *et al.* | Asia | F | 44 | Cough, dyspnea, fever | RLL | 3.7 | liver, adrenal gland pancreas | CC | CT | 0.26 |
| Yan-hui Liu *et al.* | Asia | M | 53 | Cough, hemoptysis | RLL | 5.5 | brain | CC | OP, CT, RT | 12 |
| Kamath V *et al.* | Asia | F | 37 | Weight loss | RUL | NA |  | CC | CT | 12 |
| Vegh GL *et al.* | Others | F | 30 | Cough, dyspnea hemoptysis | LLL | 4.1 |  | CC | OP, CT | 12 |
| Tajiri S *et al.* | Asia | F | 28 | Cough, dyspnea | Bilateral | NA |  | CC | OP, CT | 24 |
| Li Gong *et al.* | Asia | M | 62 | Cough, chest pain | RUL | 5 |  | CC, ASC | OP | 5 |
| Li Gong *et al.* | Asia | M | 23 | Asymptomatic | RLL | 2 |  | CC | OP | 3 |
| Li Gong *et al.* | Asia | M | 43 | Cough, hemoptysis | RUL | 5 |  | CC | OP | 7 |
| Seol HJ *et al.* | Asia | F | 19 | Chest pain | RLL | 2.5 |  | CC | OP, CT | 12 |
| Corpa Rodr guez ME *et al.* | Others | F | 31 | Cough, chest pain | RUL | 2 |  | CC | OP, CT | 42 |

Summary of literature review reporting PPC

Supplementary Table 1 (continued)

| Author | region | Sex | Age  (year) | Initial  symptom(s) | Location | Tumor size(cm) | Metastasis | Pathological  type | Therapy | Survival  (months) |
| --- | --- | --- | --- | --- | --- | --- | --- | --- | --- | --- |
| Lei He *et al.* | Asia | M | 36 | Cough, hemoptysis, fever | Bilateral | 5 |  | CC | OP, CT | 8 |
| Berthod G *et al.* | Asia | F | 32 | Abdominal pain, vaginal spotting | RUL | 1.5 |  | CC | OP, CT | 12 |
| Hadgu A *et al.* | Others | M | 48 | Cough, dyspnea, weight loss | Bilateral | NA | liver, brain | CC | OP, CT | 3 |
| Serno J *et al.* | Others | F | 31 | Positive urine pregnancy test | RUL | NA |  | CC | OP | 20 |
| Di Crescenzo V *et al*. | Others | F | 37 | Hemoptysis, dyspnea | LLL | 4 |  | CC | OP, CT | 12 |
| Qin Li *et al.* | Asia | F | 26 | Cough, chest pain | RLL | 2 |  | CC | OP, CT | 6 |
| Snoj Z *et al.* | Others | F | 35 | Chest pain | RLL | 6.4 | brain | CC, GCC | OP, CT, RT | 12 |
| Zhu R *et al.* | Asia | M | 67 | Asymptomatic | L | 9 |  | CC | OP, CT | 13 |
| Takahashi T *et al.* | Asia | M | 59 | Hemoptysis | LUL | 2.8 |  | CC, ADC | OP | 1 |
| Xuehui Pu *et al.* | Asia | M | 71 | Cough, hemoptysis | RUL, RML | 2 | lung | CC | OP | 6 |
| Kamata S *et al.* | Asia | M | 70 | Cough | RUL | 3.8 |  | CC | OP | 24 |
| Munakomi S. | Asia | F | 22 | Headache, vomit | R | NA | brain | CC | OP, CT | 2.1 |
| Rali P *et al.* | Others | F | 69 | Headache, dizziness | RUL | NA | brain | CC | OP, CT | 4.2 |
| Ma Y *et al.* | Asia | M | 71 | Cough, hemoptysis | RML | 1.6 |  | CC | OP | 3 |
| Matsukuma S *et al.* | Asia | M | 53 | Cough, chest pain | LUL | 6 | brain, lung, stomach | CC | OP, CT, RT | 15 |
| Matsukuma S *et al.* | Asia | M | 70 | Hemoptysis | R | 7 | brain, liver, adrenal gland kidney | CC, ADC | RT, other | 2 |
| Matsukuma S *et al.* | Asia | M | 77 | Tumble | LUL | 5 |  | CC, ADC | RT, other | 4 |
| Yan Peng *et al.* | Asia | M | 46 | Cough, hemoptysis, chest pain | LUL | 6 | lung, peripheral lymph node | CC | OP | 4 |
| Yan Peng *et al.* | Asia | M | 54 | Cough, hemoptysis, chest pain | LUL | 8 | lung, peripheral lymph node | CC | OP | 3 |
| Parshin VD *et al.* | Others | F | 41 | Cough, chest pain | RLL | 3.5 |  | CC | OP, CT | 4.2 |
| Juan Meng *et al.* | Asia | M | 49 | Cough, dyspnea, hemoptysis, gynecomastia | Bilateral | NA | brain | CC, SCC | CT | 9 |
| Buza N *et al.* | Others | F | 41 | Vaginal spotting | Bilateral | 9.7 | brain, liver, iliac bone | CC | OP, CT, RT | 15 |
| Wu PS. | Asia | F | 37 | Cough, dyspnea, hemoptysis, chest pain, weight loss | RLL | 16 |  | CC | OP, CT | 36 |

Summary of literature review reporting PPC

Supplementary Table 1 (continued)

| Author | region | Sex | Age  (year) | Initial  symptom(s) | Location | Tumor size(cm) | Metastasis | Pathological  type | Therapy | Survival  (months) |
| --- | --- | --- | --- | --- | --- | --- | --- | --- | --- | --- |
| Nguyen HTT *et al.* | Asia | M | 42 | Chest pain, weight loss | RUL | 15 | lung | CC, SCC | OP, CT | 6 |
| Kim JH *et al.* | Asia | F | 44 | Chest pain, fever | Bilateral | NA |  | CC | CT | 24 |
| Ben Abdallah I *et al.* | Others | F | 28 | Chest pain, amenorrhoea, vomit | RLL | 5 |  | CC | OP, CT | 72 |
| Onishi I *et al.* | Asia | F | 40 | Asymptomatic | RLL | 2.2 |  | CC | OP | 6 |

Summary of literature review reporting PPC

M, male; F, female; RUL, right upper lobe; RML, right middle lobe; RLL, right lower lobe; R, right lung; LUL, left upper lobe; LLL, left lower lung; L, left lung; OP, operation; CT, chemotherapy; RT, radiotherapy; CC, choriocarcinoma; SCC, squamous cell carcinoma, ADC, adenocarcinoma; ASC, adenosquamous carcinoma; MGCT, mixed germ cell tumours; GCC, giant cell carcinoma; ND, not described.
